# Cervical vertebral maturation and its relationship to circum-pubertal phases of the dentition in a cohort of Portuguese individuals

**DOI:** 10.4317/jced.55907

**Published:** 2019-07-01

**Authors:** Paulo Fernandes-Retto, David Matos, Margarida Ferreira, Iman Bugaighis, Ana Delgado

**Affiliations:** 1 Clínical Professor, Orthodontic Department, Faculdade Medicina Dentária. Instituto Universitário Egas Moniz, Caparica-Portugal; 2 Post graduate teaching staff at Cooperativa de ensino Superior Politecnico (CESPU), CESPU. Gandra - Paredes, Portugal; 3 Orthodontic Department, Faculdade Medicina Dentária, Instituto Universitário Egas Moniz, Caparica-Portugal; 4 BDS, DDS, MSC, PhD. Assistant professor, Orthodontic department, Faculty of Dentistry, University of Benghazi- Libya (in Sabbatical Leave). Principal investigator, Faculdade Medicina Dentária, Instituto universitário Egas Moniz, Caparica-Portugal; 5 Assistant professor, Orthodontic Department, Faculdade Medicina Dentária, Instituto Universitário Egas Moniz, Caparica-Portugal

## Abstract

**Background:**

To explore the relationship between individual skeletal maturity as assessed by Cervical Vertebral Maturity method (CVM), circum-pubertal phases of the dentition (early mixed, intermediate mixed, late mixed and early permanent) and chronlogical age in a cohort of Portuguese individuals.

**Material and Methods:**

This was a retrospective cross-sectional study. The sample comprised 300 Caucasian Portuguese subjects aged 6 to 16 years, (137 boys and 163 girls). All the participants had good quality panoramic and lateral cephalometric radiographs. Subject skeletal maturity was evaluated using the CVM method. Dental age evaluation was determined by using panoramic radiographs and dental casts. Descriptive statistics (absolute and relative frequencies) and percentages were obtained for the prevalence of the CVM stages in the various phases of the dentition and for the different ages for each of males and females.

**Results:**

Prepubertal stage 1 (CS1) was predominently observed in the early mixed dentition phase followed by the intermediate mixed dentition phase. While, the CS1 stage was found in all the other phases of tooth development. There was a wide distribution of all CVM stages in the late mixed dentition and early permanent dentition phases. While, the CS3 stage was predominantly present in both the late mixed dentition and in the early permanent dentition phases. Moreover, the chronological age did not seem to be an accurate indicator in differentiating between the pre-pubertal and the pubertal growth spurt stages.

**Conclusions:**

Early mixed dentition phase could determine with a high accuracy the prepubertal skeletal maturity stage (CS1), Furthermore, the chronological age did not seem to be an accurate indicator in differentiating between the pre-pubertal and the pubertal growth spurt stages.

** Key words:**Cervical vertebrae, cephalometric, panoramic, tooth calcification, portuguese.

## Introduction

It is generally recognized that growth and development experience phases of growth accelerations, defined as growth spurts. From this perspective, circum-pubertal growth spurts (pre-pubertal and post-pubertal) are the most significant growth periods to undertake an orthodontic treatment,(1,2) where each phase is characterized by a differential growth of the maxillary and mandibular basal bones (3). A significant body of related literature found that effective orthopaedic treatment of skeletal disharmony such as; mandibular deficiency or prognathism, maxillary transverse constriction and skeletal deep bite, critically relies on the level of skeletal maturation throughout treatment (4,5). Furthermore, accurate assessment of skeletal age is essential for the evaluation of the aetiology of craniofacial problems, determining the diagnosis, selecting the most appropriate orthodontic treatment, and aiding in the evaluation of treatment outcome including post treatment relapse if present (3,6) It has become ever more established that optimal timing for dentoskeletal orthopedic treatment could be as decisive as the selection of the treatment of choice (4,5).

There are several non-invasive biological indicators used for the assessment of the stage of individual’s skeletal maturity such as; chronological age, increased body height (7), menarche voice changes (8), and tooth eruption (7,9) It has been demonstrated that chronological age offers a weak biological ground for growth assessment (10). Also, even though, peak growth rate in height is the most reliable approach to predict the stage of skeletal growth, it has a limited use in estimating the percentage and rate of the remaining growth (11). Menarche and voice change (8) have been demonstrated to be inaccurate for predicting pubertal growth spurt (12). Furthermore, tooth emergence could be affected by local or general factors such as space defeciency or systemic diseaes (9). Therefore, panoramic, hand and wrist and lateral cephalometric radiographs have been recommended to be used in evaluating skeletal maturity (4,13,14). Panoramic radiographs are used to examine and correlate the stages of crown and root formation as well as tooth emergence with skeletal development, taking into consideration the limitations of relying on timing of tooth eruption (9,15). A commonly used dental age evaluation method (deciduous dentition, early mixed dentition, late mixed dentition and early permanent dentition stages) have been frequently used in the assessment of class II and class III treatment protocols at earlier versus later phases of dental development (16-18). Franchi *et al.* (19) observed that early mixed dentition phase had a strong diagnostic value for the determination of prepubertal cervical maturity (CS1), while, intermediate and mixed dentition phases showed low diagnostic value for the same prepubertal phase.

Hand and wrist radiographs had been widely used to visually evaluate the stages of bone ossification in hand, wrist and fingers (2,20). However, there have been growing concerns related to a further exposure to radiation by the acquisition of additional radiograph. Subsequently, the British Orthodontic Society has affirmed that their use to anticipate the beginning of the pubertal growth spurt are not recommended (21).

Accuratily extracted lateral cephalometric radiographs, showing the cervical spine, have been increasingly used to assess and correlate the level of Cervical Vertebral Maturation (CVM) with the stage of skeletal maturity (13,22,23). CVM technique has the advantage of using the same cephalometric radiographs that are requested routinely in orthodontics (3,5,21). A number of studies have demonstrated that the CVM method is a reliable indicator for determining the circum-pubertal growth phases (3,12,13,24). Skeletal maturity stages are determined by visualizing the postero-anterior evolving morphological changes of the lower edge of the cervical vertebrae during growth; from trapezoid tapering to rectangular with a greater horizontal width, square and then rectangular with an increased vertical length (4,5,13).

A number of studies were undertaken on different population cohorts using the CMV method and dental maturity phases such as; Italians, (4,13) Iranian females (25), Greeks (26), Thais (16), Americans (3), British (22), Chinese (2) and Turkish (18). However, discrepancies in the level of association between skeletal, dental and chronological ages have been observed among the published studies (27). These disparities might be due to differences in sample size, climate change, and/or racial variations (28). Up to date, the relationship between skeletal, dental and chronological ages in Portuguese population have not been explored. Therefore, the aim of this preliminary investigation was to describe and explore the relation between individual skeletal maturity as assessed by CVM, the circum-pubertal phases of the dentition (early mixed, intermediate mixed, late mixed and early permanent) and the chronlogical age in a cohort of Portuguese individuals.

## Material and Methods

This was a cross-sectional retrospective investigation which included 300 Caucasian Portuguese subjects aged 6 to 16 years, (137 boys and 163 girls) over a two year period. This cohort was selected from the pre-orthodontic records of a greater sample (425) who were attending the orthodontic clinic of the Instituto Superior de Ciências da Saúde, Egas Moniz. Ethical approval was granted from the Ethics Committee of Egas Moniz - Cooperativa de Ensino Superior and written consents were obtained from the included subjects prior to undertaking this research study.

The inclusion criteria comprised Caucasians Portuguese subjects aged between 6 and 16 years. All participants had no previous orthodontic treatment; none had a cleft lip and palate, malformations of the cervical vertebrae, or any other craniofacial anomaly. Subjects with hypodontia, supernumerary teeth, and early loss of deciduous dentition due to trauma or caries were excluded from the study. All the participants had good quality panoramic and lateral cephalometric radiographs. Anatomical structures particularly cervical vertebrae were clearly visible on lateral cephalometric radiographs. The study models of the participants were inspected to ensure their good quality and suitability for the present research.

Dental Analysis 

The sample comprised subjects in the four dentition phases. The evaluation of both the dental casts and panoramic radiographs were undertaken according to the following criteria (19);

1. Early mixed dentition: shedding of the deciduous incisors, eruption of the first permanent molars and permanent incisors.

2. Intermediate mixed dentition: permanent incisors and first molars are fully erupted, presence of deciduous teeth in the buccal region (deciduous canine, first molar, and second molar).

3. Late mixed dentition: shedding of the deciduous canines and molars, eruption of the permanent canines and premolars.

4. Early permanent dentition: presence of all permanent teeth (possible presence of second molars; absence of third molars).

-CVM Analysis 

**Figure 1:**
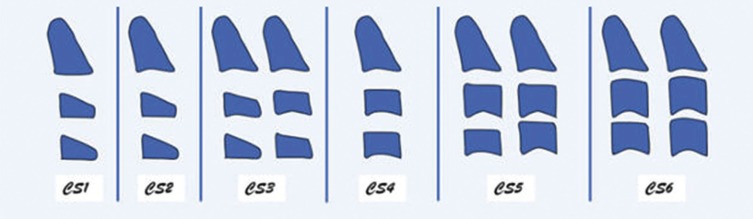
Schematic illustration of the six Cervical Vertebral Maturation (CVM) .

CVM is a reliable method used for the assessment of skeletal maturity through visualizing the morphology of cervical vertebrae on each cephalogram (5,19). Baccetti and co-workers5 proposed the CVM six stages as follow; (CS1 to CS6); CS1 and CS2 stages occur before the growth spurt, while the growth peak takes place amid CS3 and CS4, also, CS5 and CS6 stages are observed following the growth peak ( Table 1, Fig. 1). The determination of each stage is based on two categories of variables.

**Table 1:**
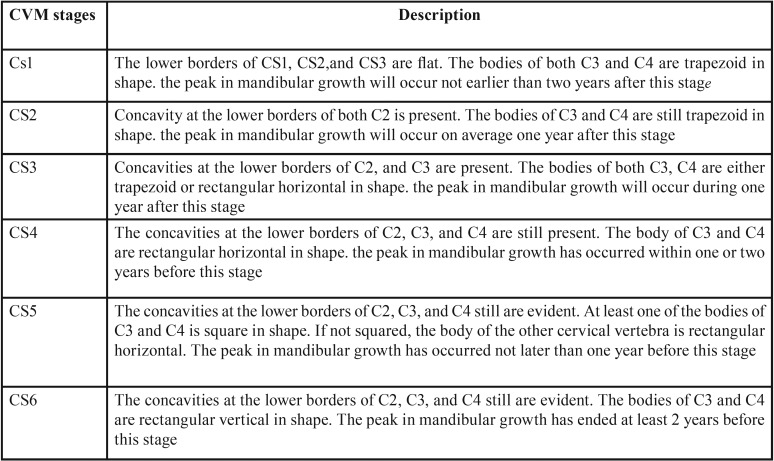
Description of the six stages of cervical vertebral maturation (CVM).

1- Occurrence or absence of a concavity at the lower margin of C2, C3 and C4;

2- Morphological variations of the body of C3 and C4:

a- Trapezoid (the superior vertebral margin is tapered postero-anteriorly);

b- Rectangular horizontal (equal heights of the anterior and posterior vertebral margins while; the superior and inferior margins are longer than the anterior and posterior margins);

C- Squared: the four margins (superior, inferior, anterior, posterior) are equal in length;

D- Rectangular vertical (the posterior and anterior margins are longer than the superior and inferior margins).

Statistical Analysis

Statistical analysis was undertaken using IBM SPSS Statistics (version 15). Percent agreement and kappa statistics were computed for the assessment of intra-examiner and inter-examiner agreement.

Descriptive statistics (absolute and relative frequencies) were obtained for the prevalence of the CVM stages in the various phases of the dentition and for the different ages for each of the males and females.

-Calibration

 The examiners who undertook this study were trained and calibrated by an expert in this method. A 100 previously analyzed and classified cephalograms were re-analyised by the three examiners after a two-week interval. The inter-examiner agreement was 95.0 % with a Kappa of 0.94.

The cephalograms, panoramic radiographs and dental casts of each participant were visualised and appraised independently by the three examiners, two weeks apart from each other, with an average intra-examiner agreement of 99%, and with an average inter-examiner agreement of 94.0% with a Kappa of 0.917. The examiners while evaluating the phases of the dentition and dental casts were blinded as to CVM stages, as well as the chronological age and vice versa. There was a disagreement between the researchers in the classification of the CVM stages in 18 cephalograms. This disparity was resolved by taking a majority of two opinions against one.

## Results

The present cohort (300 subject) comprised 137 males (45.7%) and 163 females (54.3%).  Table 2 and  Table 3 illustrate sex distribution in the different dentition phases and CVM stages, respectively.

**Table 2:**
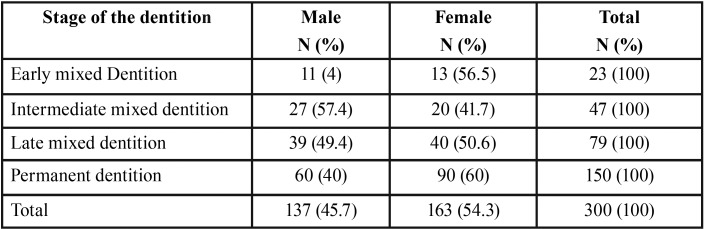
Sex distribution (in numbers [N] and percentage [%]) among the different stages of dentition.

**Table 3:**
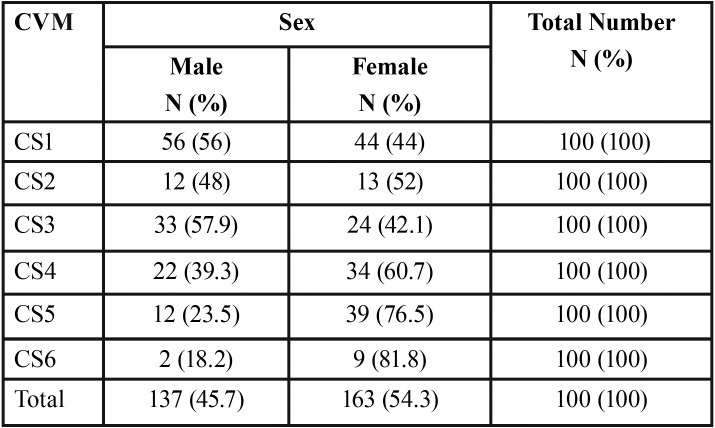
Sex distribution (in numbers [N] and percentage [%]) among the different cervical vertebral maturation stages (CVM).

 Table 4 illustrates that in their early mixed dentition, the subjects were predominantly observed in CS1; 95.8% of the subjects in the early mixed dentition were in the CS1 stage. However, the CS1 stage was found in all the other phases of tooth development and it was more prevalent in the intermediate mixed dentition phases (39.3% in males and 13% in females) and the late mixed dentition (30.4% in males and 34.1% in females). Moreover, there was a wide distribution of all CVM stages in the late mixed dentition and early permanent dentition phases. The CS3 stage was predominantly present in the late mixed dentition (30.3% in males and 33.3% in females) and early permanent dentition phases (30.3% and 66.7% in males and 33.3% and 54% in females).

**Table 4:**
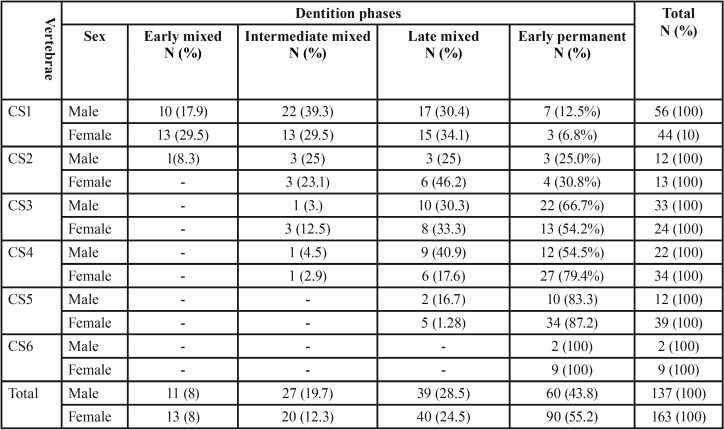
The prevalence (in numbers [N] and percentage [%]) of Cervical Vertebral Maturation (CVM) in the various dentition phases for each dental phase.

 Table 5 presents the descriptive statistics concerning chronological age and CVM stages. These figures present a gradual increase in the presence of the CVM stages with age. The CS1 and the CS2 stages could be observed in a wide range of age. The CS3 stage is more frequent between the ages of 11 and 13 and the CSV4 stage is more common between the ages of 12-14 years.

**Table 5:**
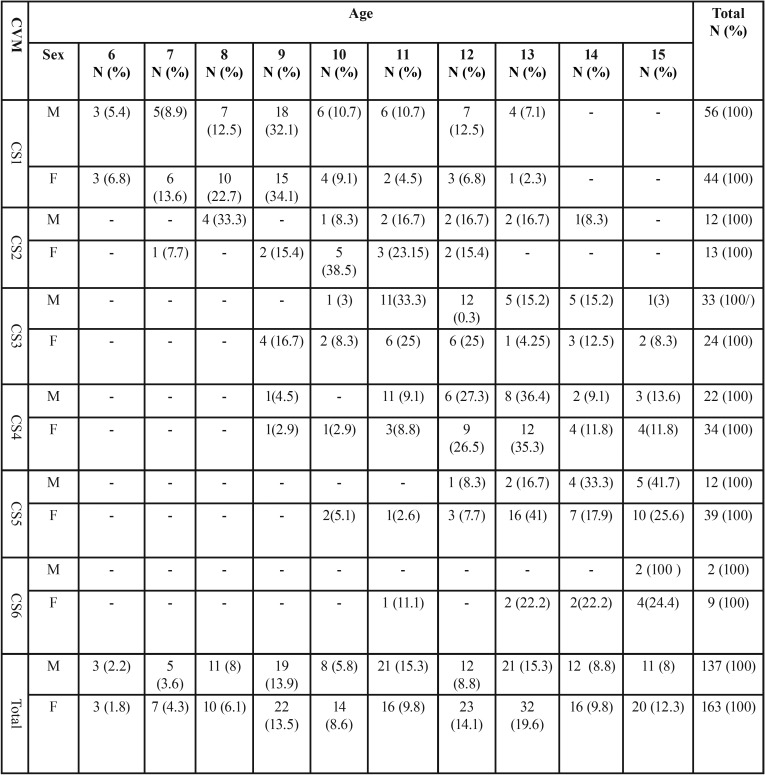
Distribution of chronological ages (in numbers [N] and percentage) for Males (M) and Females (F) grouped by cervical maturation vertebra stages (CVM).

## Discussion

This retrospective cross-sectional study was the first to be undertaken in a group of Portuguese subjects for the assessment of the CVM, its relation with the circum-pubertal phases of the dentition and the participant’s chronological age.

CVM method proved to be a reliable biological indicator for the assessment of the stages of skeletal maturity (3,4,13,16,29). Therefore, this method has been recommended to be used in a wide range of research and in clinical settings (3,4,13,16,29). A number of similar studies using the CMV method and dental maturity phases were undertaken (9,16,18,19,30). Majority of those studies employed a correlation analysis to reliably evaluate the diagnostic relevance of dental development and chronological age for the determination of skeletal maturity (16).

 Table 4 and  Table 5 reveal that that the prevalence of the examined sample did not cover all the groups impeding the conduction of inferential statistical analysis, therefore, descriptive statistics were the only employed analyses.

The relationship between the onset of puberty and dental maturation is not well defined. Studies have shown that the correlation between tooth mineralization and other parameters of physical development is generally low (9,10,14,16). However, it has been observed in the present study that early mixed dentition phase is useful for the determination of CS1 stage. Similar findings were stated by a number of comparable research studies with larger sample size. A number of research studies recommended that this phase could be the ideal time to start orthopaedic treatment aiming at modifying skeletal growth (such as facemask treatment and maxillary expansion (3,4,13). In other words, the progressive phase identified by shedding of the deciduous incisors and eruption of permanent incisors in addition to first molars is a strong indicator of the pre-pubertal stage of skeletal maturity.

The intermediate mixed dentition phase continued to indicate that 85.4% of the participants were at a pre-pubertal stage of skeletal maturity (72.9% at CS1 and 12.5% at CS2). Contrarily, late mixed dentition phase did not seem to be helpful for defining the beginning of the pubertal growth spurt (CS3). During the late mixed dentition phase, while, permanent canines or premolars are erupting, around one third of the individuals were at the beginning of their pubertal growth spurt (CS3 stage), and roughly one third were at their pre-pubertal growth stage (CS1 and CS2). The remaining subjects (around one third) existed in the post-pubertal stage of skeletal maturity (CS4). Furthermore, the results revealed that the early permanent dentition phase is not a reliable indicator of the peak of skeletal maturity and that subjects could be observed in all the skeletal maturity stages (CS1-SC6); almost one quarter of the subjects in their early permanent dentition could demonstrate either the beginning of the pubertal growth spurt (CS3) or a post peak stage (CS4). Also, around one tenth of the subjects could be observed at each of the pre-pubertal stages of skeletal maturity (CS1 and CS2) and at the post-pubertal growth spurt stage (CS5 and CS6). These findings demonstrate that late mixed and early permanent dentition phases are poor identifiers of the commencement of the pubertal growth spurt which appear to be in agreement with the results of similar studies (3,29).

The distribution of age ranges, during the onset of pre-pubertal growth (before CS3) is wide, particularly in male subjects ( Table 5) Males under 10 years old and females under 9 years old were never observed in stage CS3 or above. This finding indicates that CS1 might identify the pre-pubertal stage. However, subjects in CS3, which corresponds to the beginning of the growth peak, were found from 9 to 16 years, but more commonly between 11 and 13 years (63.6% in males and 50% in females). Stage CS4 was more frequent in subjects between 12 and 14 years (62.5%) and with similar frequency distribution in both sexes (67.7% in males and 61.8% in females). These findings show that, in the present cohort, the chronological age was not an accurate indicator in differentiating between the pre-pubertal and the pubertal growth spurt stages. Several comparable studies reached the same conclusion (3,4,19). While, other researchers, reported a higher association between skeletal age and chronological age (14). These described differences might be attributed to the different examined racial backgrounds and to the different undertaken statistical analysis. However, CVM method has been shown to be a reliable developmental indicator in detecting somatic maturation (4,6).

## Conclusions

In the present cohort early mixed dentition phase could determine with a high accuracy the prepubertal skeletal maturity (CS1), whereas, the intermediate mixed dentition phase had a low diagnostic value for the same pre-pubertal stage (CS1). Neither the late mixed dentition nor the early permanent dentition stages are reliable indicators for the beginning of the pubertal growth spurt. Moreover, the chronological age did not seem to be an accurate indicator in differentiating between the pre-pubertal and the pubertal growth spurt stages.
